# The Anti-Vaccinators and the Royal Commission

**Published:** 1889-06-29

**Authors:** William Tebb

**Affiliations:** Devonshire Club, St. James's, London


### Editor's Letter-Box.

Our Correspondents are reminded that prolixity is a great bar to publication, and that brevity of style and conciseness of statement greatly facilitate early insertion.]

### THE ANTI-VACCINATORS AND THE ROYAL COMMISSION.

Sir,—In his letter to the *Times*, 10th inst., Dr. Edwardes is of opinion that my complaint of the constitution of this Commission is unreasonable, and, in support of his contention, informs us that the German Vaccination Commission of 1884 comprised eighteen members, of whom a sixth only were opposed to compulsory vaccination. I do not dispute his facts, but I fail to see why a tribunal so obviously unfair, and which has not had the smallest effect in arresting the agitation against the vaccination laws in the German Empire, should be cited for our imitation. A correspondent, the editor of a German medical journal, whose letter dated January 16, 1885, referring to this Commission, lies before me, says: "The proceedings of the Vaccination Commission have been a farce; the anti-vaccinators were not allowed to lay down their points of view. Better have no Commission at all than one with three anti-vaccinators and twelve pro-vaccinators, and a president entirely under the influence of the latter."

When Mr. Ritchie agreed to concede a Royal Commission, it is clear that one of a different character and scope to that appointed was expected, as the debate on the 5th April shows. Dr. R. Farquharson, in the interest of vaccination, asked that it should be "full, elaborate, and comprehensive in character." Sir Lyon Playfair said "there should be a full and impartial enquiry," and he had no doubt the right hon. gentleman (Mr. Ritchie) would constitute it with perfect fairness. The Right Hon. James Stansfeld, who testified as an ex-President of the Local Government Board that "vaccination was to his own knowledge attended with danger to health and life," urged that the enquiry should be "full, free, and satisfactory to the public who are most concerned." In the course of his several replies, Mr. Ritchie allowed that public opinion required a thorough investigation into the whole question, and that the Commission should be composed of gentlemen whose opinions will carry the greatest possible weight in all quarters, and that it would be undesirable to fetter the question. Several members expressed their warm gratitude to Mr. Ritchie for the magnanimous way in which he had conferred the Commission, and the announcement the next day was welcomed with lively satisfaction all over the country. Dr. Edwardes considers that the fulfilment of these promises would be a gratuitous concession to the clamour of a few, which shows that he is ill-informed as to the depth and extent of the insurrection against the Vaccination Acts, or of the acute sense of injustice and unmerited suffering to which they give rise. William Tebb.

Devonshire Club, St. James's, London, June 15, 1889.

[This letter, from the President of the Anti-Vaccination Society, is exactly what might have been expected. Lord Herschell up till now has been held a man of fearless honesty and independence. He is, too, politically, in opposition to those who have asked him to be chairman of the Vaccination Commission. But no sooner does he accept the post than the abuse of him is begun, Mr. Tebb leading the way by adopting the opinion of an anonymous German anti-vaccinator (introduced with a flourish as "the editor of a German medical journal"), that Lord Herschell is so dishonest and so weak-minded as to be willing to place himself "entirely under the influence" of a section of his Commissioners. So, too, the Vaccination Inquirer has been for months urging and begging that a Commission should be appointed. How is the boon accepted? With an ungrateful snarl that is useful only as indicating to a nicety the character of the recipients. Even when in absolute ignorance as to the composition of the Commission, the Inquirer said, "Let us be under no illusion. This Commission is not designed for our advantage. . . . It is intended to afford ground for strengthening the law. . . . We do not imagine that any Commission can permanently arrest truth, . . . but it is quite possible to set it back for a time by stimulating and confirming prejudice against it. That such is the calculation we are left without doubt. . . . The Commission has been conceded at this time to serve the ends of the medical officials of the Local Government Board, and how skilful and unscrupulous they are, and how like wax they moved Mr. Ritchie to their ends, etc., . . ." Mr. Tebb mentions that in the course of the parliamentary debate "several members expressed their warm gratitude to Mr. Ritchie for the magnanimous way in which he had conferred the Commission." What does he think of the "warm gratitude" of the organ and mouthpiece of his society? It would appear that the only kind of Commission which would have pleased the opponents of vaccination would have had to be so constituted that a verdict against vaccination must have been guaranteed from the start. In the scientific world there is not one man in a thousand opposed to vaccination, while of the Commissioners almost 20 per cent. are admitted, even by Mr. Tebb's German editor, to be anti-vaccinators. In complaining of this proportion, Mr. Tebb pays a poor compliment to Messrs. Bradlaugh, Picton, and Collins. But the fact is that he and his following are beginning to get alarmed at the possible consequences of the granting of their own petition, and so they are now doing their best to discount the value of the overwhelming evidence of the benefits of vaccination which, they have a shrewd suspicion, will be laid before the Royal Commission.—Ed.]

